# The Properties and Functions of Glial Cell Types of the Hypothalamic Median Eminence

**DOI:** 10.3389/fendo.2022.953995

**Published:** 2022-07-27

**Authors:** Richard W. Clayton, Robin Lovell-Badge, Christophe Galichet

**Affiliations:** Laboratory of Stem Cell Biology and Developmental Genetics, The Francis Crick Institute, London, United Kingdom

**Keywords:** hypothalamus, pituitary gland, median eminence (ME), oligodendrocyte precursor cells (OPCs), NG2 glia, microglia, astrocytes, tanycytes

## Abstract

The median eminence (ME) is part of the neuroendocrine system (NES) that functions as a crucial interface between the hypothalamus and pituitary gland. The ME contains many non-neuronal cell types, including oligodendrocytes, oligodendrocyte precursor cells (OPCs), tanycytes, astrocytes, pericytes, microglia and other immune cells, which may be involved in the regulation of NES function. For example, in mice, ablation of tanycytes (a special class of ependymal glia with stem cell-like functions) results in weight gain, feeding, insulin insensitivity and increased visceral adipose, consistent with the demonstrated ability of these cells to sense and transport both glucose and leptin, and to differentiate into neurons that control feeding and metabolism in the hypothalamus. To give a further example, OPCs in the ME of mice have been shown to rapidly respond to dietary signals, in turn controlling composition of the extracellular matrix in the ME, derived from oligodendrocyte-lineage cells, which may contribute to the previously described role of these cells in actively maintaining leptin-receptor-expressing dendrites in the ME. In this review, we explore and discuss recent advances such as these, that have developed our understanding of how the various cell types of the ME contribute to its function in the NES as the interface between the hypothalamus and pituitary gland. We also highlight avenues of future research which promise to uncover additional functions of the ME and the glia, stem and progenitor cells it contains.

## Introduction

The neuroendocrine system (NES) is a vital multi-organ and hormonal system controlling mammalian physiology, and NES dysfunction has profound effects on human health. Hormones secreted by the pituitary gland direct the function of end-target organs, such as the thyroid, liver, gonads, mammary and adrenal glands, thereby regulating fundamental processes like metabolism, reproduction, stress response, lactation and growth (1). Production and secretion of pituitary hormones is controlled by the hypothalamus, which secretes its own array of neuronal-derived hormones (neurohormones) that are secreted either into the hypophyseal-portal blood vessels in the ventral hypothalamus (for the anterior pituitary gland), or directly into the blood stream at the level of the posterior pituitary gland (or *pars nervosa*) (1).

The hypothalamic neurons that synthesise and secrete neurohormones extend axons that pass through a ventral sub-structure of the hypothalamus called the median eminence (ME), which is located at the base of the third ventricle (1). Fenestrated capillaries in the ventral portion of the ME (1), combined with the presence of tanycytes dorsally, mean that the ME resides outside the blood brain barrier (BBB), and that hormones and other signals that can diffuse into the ME from the peripheral circulation cannot diffuse into the cerebrospinal fluid (CSF) or the wider hypothalamus (2). This property of the ME is particularly interesting in the context of targeted drug delivery for treatment of neuroendocrine disorders. Conceivably, druggable cellular targets at the level of the ME could be specifically targeted by modifying drugs in such a way that they cannot cross the tanycytic-brain barrier, thus sparing the rest of the brain.

While the ME is the termination point for the axons of many hypothalamic neurons (1), it also contains dendrites which extend from neurons in the arcuate nucleus (ArcN) of the hypothalamus. For example, leptin receptor-expressing dendrites extend into the ME and detect levels of circulating adipose-produced leptin in the extracellular milieu. The leptin system regulates feeding behaviour based on body fat content, and degradation of these dendrites in mice is associated with leptin insensitivity, overeating and increased adiposity (3).

**Figure 1:**
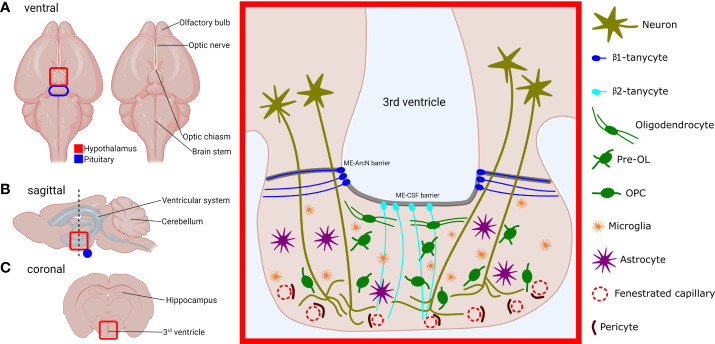
Location, structure and cell types of the murine median eminence (ME). Locations of the hypothalamus (red) and pituitary (blue) are outlined, in the ventral **(A)**, sagittal **(B)** and coronal view **(C)**. Red-boxed area on the left represents the red-highlighted area in the coronal plane **(C)**, and shows the structure of the ME and its constituent cell types. OL, oligodendrocyte; OPC, oligodendrocyte precursor cell. Neurohormone-producing neurons project from the hypothalamus and abrogate in the ventral portion of the ME which contains fenestrated capillaries. Secreted hormones are then carried through the portal vasculature to the anterior pituitary gland. The 3^rd^ ventricle at the ME is lined with tanycytes. β1-tanycytes project laterally and are thought to isolate the ME milieu from the rest of the hypothalamus. β2-tanycytes project ventrally and junctions between their cell bodies prevent diffusion of substances from the ME to the CSF and vice versa. Myelinating oligodendrocytes are predominantly found in the dorsal section of the ME, which contains axons that project to the posterior pituitary. OPCs, microglia, and astrocytes are found throughout the ME body.

Moreover, aside from neuronal and vascular components, the ME contains many different glial cell glial cell types (**Figure 1**), each with distinct functions (**Table 1**). Single-cell RNA sequencing (scRNAseq) of dissected MEs has demonstrated that the ME contains oligodendrocytes, oligodendrocyte precursor cells (OPCs), microglia, astrocytes, ependymal cells, tanycytes, endothelial cells and other vascular and leptomeningeal cell types (12) (**Table 1**). Moreover, at least some of these cell types can also sense and respond to peripheral signals, such as blood glucose levels (16). Given the important location of the ME within the NES, it follows that any cell type resident in the ME has the potential to exert profound regulatory control over the NES. In this review, we will visit key glial cell types and describe and discuss recent evidence demonstrating functional roles of these cell types in the ME.

**Table 1:** Cell types of the median eminence (ME) and their positions, marker genes and known functions.

Glial cell type	Location in median eminence (ME)	Key marker genes	Known functions in ME	Key references
β1-tanycyte	Cell bodies at base of 3rd ventricle (lateral), extend processes laterally into the hypothalamus.	*Rax, Vimentin, Nestin*	Possible role in forming ME-ArcN barrier.	(4)
β2-tanycyte	Base of 3rd ventricle (medial), extend processes ventrally towards portal blood vessels and meninges.	*Rax, Vimentin, Nestin*	Glucose and leptin transport, ME-CSF barrier, stem cell potential, regulation of leptin sensing, physical interaction with GnRH+ neurons.	(4–11)
Astrocyte	Throughout the ME.	*Gfap, Agt*	Not known, but well described role in modulation of neurons in wider hypothalamus.	(12–14)
Oligodendrocyte (OL)	Predominantly in the dorsal portion of the ME.	*Mag, Mog, Apc, Plp1*	Not known, but myelin deposited in dorsal portion of ME.	(12)
Oligodendrocyte progenitor cell (OPC)	Throughout the ME.	*Pdgfrα, Cspg4 (Ng2)*	Physical interaction with leptin receptor-expressing dendrites required for leptin sensing. Progenitors for oligodendrocyte lineage.	(3, 12)
Pre-OL (or differentiating OPC)	Throughout the ME at early stages of differentiation, to predominantly dorsal at later stages.	*Bmp4* (early), *Tcf7l2* (late)	Expression of Tenascin-R and ECM components comprising peri-neural nets, degradation of which increases food intake/weight gain in mice.	(12)
Microglia	Throughout the ME.	*Iba-1 (Aif1)*	Microglia-mediated inflammatory status may regulate leptin sensing.	(3, 15)

## Tanycytes

Tanycytes are a specialised type of ependymal cell that line the walls of the third and fourth ventricles. Tanycytes are highly polarised, and while their cell bodies located within the ependymal cell layer, tanycytes also extend long processes into the hypothalamus and ME. Tanycytes can be divided into several sub-groups, each with different properties regarding stem cell- and barrier-forming capabilities (17). The ME contains β1-tanycytes and β2-tanycytes (**Figure 1**), while α-tanycytes can be found lining the 3^rd^ ventricle in the higher parts of the hypothalamus (18).

Tanycytes have been the subject of particular interest given their reported stem cell capabilities. Lineage tracing of tanycytes demonstrates that they can differentiate into neurons and glia and it has been hypothesised that regulation of the stem cell potential of tanycytes would be a way of regulating the output of the ME and hypothalamus. Furthermore, loss of tanycytic stem cell potential with age or disease may explain reductions in NES function (17).

The stem cell potential of tanycytes continues to be a field of constant investigation. Tanycytes have been shown to exit quiescence and proliferate following mechanical injury to the ME in an *Igf1r*-dependent manner; although the differentiation of tanycytes into neurons and other cell types is limited in this system (5). Lineage tracing of β2-tanycytes based on *Fgf10* expression shows that they can contribute neurons to the hypothalamic nuclei, chiefly the ArcN (19). Interestingly, selective expression of oncogenic *Braf* in β2-tanycytes leads to the formation of craniopharyngoma-like tumours, suggesting that these tanycytes are the cell type of origin for this type of cancer (5). X-ray irradiation of the ME reduces the number of BrdU+ newly-born neurons in the ME, suggesting reduced neurogenesis from tanycytes following irradiation, and this is associated with reduced body weight and increased energy expenditure in mice (6), however, these results could also be due to loss of OPCs following irradiation (3).

It is also known that tanycytes have a barrier-forming function. β2-tanycytes (Rax-expressing (5);) form tight- and adherens junctions between their cell bodies and also express ZO-1, thereby forming a barrier separating the ME from CSF (ME-CSF barrier) (2, 18) (**Figure 1**). The apposition of the cell bodies of tanycytes to the ventricles exposes tanycytes to molecules contained within the CSF, and it has been shown that tanycytes can shuttle large molecules from the CSF to the ME and ArcN (20). Tanycytes may therefore represent a conduit through which CSF-borne signals could regulate the function of the hypothalamus. It is also clear that diffusion of substances laterally from the ME into the ArcN is similarly restricted (ME-ArcN barrier) (7) (**Figure 1**), but the nature and purpose of this barrier is less well understood. Extensive interdigitation of cellular processes and adherens junctions between β1-tanycytes likely comprises at least part of this barrier (4). Together, the tanycytic ME-CSF barrier and ME-ArcN barrier, combined with fenestration of portal capillaries in the ME ensure that, uniquely for the hypothalamus, the ME remains outside the BBB.

Another field of intensive research has been the regulation of tanycytes by dietary signals. For example, fasting results in increased neurogenesis from nestin-expressing tanycytes in p19 female mice (6, 21). Fasting also induces tanycytic-VEGF expression that increases capillary fenestration in the ME and also increases permeability of the ME-ArcN barrier (22). Mice on a high-fat diet show ultrastructural changes in tanycytes including lipid accumulation, organelle degradation and reduced junction formation (23) that aligns with reports of increased permeability of the ME-CSF barrier in mice following hyperglycaemia (7). Loss of Igf1r signalling in tanycytes impairs the tanycytic ability to proliferate following injury to the ME (5) and, in terms of differential gene expression, tanycytes are the second most responsive to a fasting-refeeding paradigm in mice, after astrocytes (12). Moreover, tanycytes also express glucose transporter proteins (7, 24, 25), though different glucose transporter proteins have been demonstrated in β1-tanycytes and β2-tanycytes (7, 25).

While the functional consequences of dietary-associated changes in tanycytes are not entirely clear, diphtheria toxin-mediated genetic ablation of β2-tanycytes reduces ME-CSF barrier function and increases adiposity under thermoneutral conditions (8), suggesting a possible effect on either leptin-sensing or metabolism. Indeed, both α- and β-tanycytes have been shown to express express leptin receptor (LepR) and generate calcium waves in response to leptin (9), and appear to be able to shuttle leptin to the medio-basal hypothalamus in a manner dependent on ERK-signalling (9, 26). Loss of tanycytic LepR expression results in not only increased feeding behaviour and adiposity but also insulin insensitivity, which may result from changes in autonomic innervation to the pancreas (9).

Interestingly, it has been shown that α-tanycytic conversion of glucose into lactate, and subsequent transport of lactate, drives activity in adjacent proopiomelanocortin (POMC)-expressing neurons in the ArcN (27). Infusion of α-tanycytes with lactate drives neuronal activity in POMC neurons, and blockade of lactate synthesis in α-tanycytes reduces neuronal activity and increases mouse body weight and feeding. Inhibition or loss of tanycytic gap junctions abrogates this process (27). It would be interesting to see whether β-tanycytes of the ME are also capable of a similar function, which are more exposed to glucose and other signals given that the ME is outside the BBB (7). Alternatively, perhaps β-tanycytes collect and transport glucose to α-tanycytes, either *via* the CSF or *via* tanycytic gap junctions, as part of a system that regulates feeding behaviour in response to glucose levels.

Furthermore, a response of tanycytes to dietary signals appars to be conserved across different species (28) and it is also known that tanycytes regulate the gonadotropic system by interacting with and regulating gonadotropin-releasing hormone-secreting (GnRH+) nerve terminals in the ME (22, 29, 30). Tanycytes may therefore represent a nexus through which dietary signals, such as food availability, may regulate reproduction and the reproductive endocrine axis more generally.

Indeed, with regards to the gonadotropic system, tanycytes express semaphorin7a (Sema7a) in an oestrous cycle-dependent manner. Ablation of the receptor for Sema7a (plexinC1) leads to abnormal innervation of the ME and subsequently reduced fertility in mice (29). Tanycytes also appear to respond to changes in circulating prolactin (PRL) levels, by increasing the levels of pSTAT5, which was associated with reduced infiltration of Evan’s blue dye into the ME. This suggests that PRL-sensing by tanycytes can regulate permeability of the ME (31).

Finally, recent evidence has shown that tanycytes not only regulate the gonadotropic axis, but also other hormone pathways within the NES. Endocannabinoids are neuromodulatory lipids (e.g. 2-Arachidonoylglycerol or 2-AG), and tanycyte-derived endocannabinoids appear to inhibit thyroid-stimulating hormone (TSH) release from thyrotropic neurons, as tanycyte-specific deletion of diacylglycerol lipase alpha, a key enzyme in endocannabinoid synthesis, reduces 2-AG synthesis and increases TSHβ expression (32, 33). Tanycytes also regulate the thyrotropic axis by dynamically regulating the size of tancytic endfeet in contact with thyrotropic neurons, and by secreting ectopeptidases, such as pyroglutamyl peptidase II (PPII), which can degrade hypothalamic Thyrotropin-releasing hormone (TRH) (34, 35).

## Oligodendrocytes

Oligodendrocytes are glial cells found throughout the CNS. White matter is so-named due to the presence of myelin, which is synthesised and secreted by oligodendrocytes. Myelin sheaths enwrap neuronal axons and expedite neurotransmission by facilitating rapid saltatory conduction. In the ME, oligodendrocytes reside predominantly in the dorsal portion, near the base of the 3^rd^ ventricle, and myelin and myelin-associated proteins also appear to be restricted to this part of the ME (12) (**Figure 1**). This localisation might suggest that myelination is only functionally important for axons projecting to the *pars nervosa* of the posterior pituitary, and not for those that terminate in the ventral ME to regulate anterior pituitary secretions. With regards to the relative importance of myelin to the anterior and posterior pituitary, a future experiment might involve infusion of the 3^rd^ ventricle with cuprizone, thus demyelinating the median eminence. Subsequently, levels of anterior and posterior pituitary hormones could then be investigated, although the ME-CSF barrier could present an obstacle to infusing the ME with cuprizone or other drugs (18).

Moreover, while there are some clinical associations between levels of anterior pituitary hormones and demyelinating diseases, such as multiple sclerosis (36–38), it is difficult to disentangle these links from possible effects of these diseases on oligodendrocyte precursor cells (OPCs) and pre-OLs, which may serve their own functions in the ME that are independent of myelination (see section: OPCs and pre-OLs). Interestingly, dietary signals have been shown to dynamically regulate oligodendrocyte differentiation in the murine ME (12). However, at least in the short-term context of a 16-hour fast: 2-hour refeeding paradigm, there is no effect on myelination in the ME, despite the promotion of an oligodendrocyte differentiation gene expression programme (12). Perhaps effects on myelination would be manifest over more long-term dietary experiments, in which case, a link could be drawn between dietary signals, myelination, and the function of oxytocin-producing axons that pass through the dorsal ME, which are also thought to control feeding behaviour (39).

## OPCs and Pre-OLs

Oligodendrocyte precursor cells (OPCs; also known as NG2-glia) are a type of proliferative glia that give rise to oligodendrocytes by differentiation through intermediate cell types referred to as differentiating OPCs or pre-OLs (pre-oligodendrocytes) (12, 40, 41). As one of the most proliferative cell types in the brain (42), OPCs account for the large majority of mitotic cells in the ME; most of the remainder being accounted for by microglia (3). This proliferation would suggest that there is a constant turnover of the oligodendrocyte lineage cells in the ME. Indeed, targeting of proliferating cells by irradiation or experimental use of chemotherapeutics leads to a rapid loss of OPCs in the ME (3). As mentioned above, the reason for this seemingly constant turnover is not yet clear but, nonetheless, it is evident that OPC differentiation in the ME is dynamic and changes in response to physiological stimuli (12). Interestingly, engulfment and destruction of OPCs by microglia in the mouse brain has been shown to be a method of dynamically regulating myelination during development (43, 44), and perhaps the same is true in the ME, with OPC cell numbers resulting from a balance of proliferation and phagocytosis by microglia.

Aside from differentiating into oligodendrocytes, it is increasingly clear that OPCs serve several functions independent of their role as progenitors (45). In the ME, for example, OPCs interact with LepR+ dendrites, and ablation of OPCs in the ME by radiation, chemotherapeutics, or genetic techniques results in a collapse of this leptin-sensing dendritic network, culminating in overeating and obesity (3). The close physical relationship between OPCs and LepR+ dendrites suggests that OPCs provide mechanical support, but trophic support through chemical messengers could also be possible. Indeed, OPCs in the cortex have been shown to secrete FGF2, loss of which results in glutamatergic dysfunction and depressive-behaviours in mice (46). Interestingly, in response to a short-term fasting and re-feeding paradigm, rapid differentiation of OPCs was shown to be associated with remodelling of glutamatergic vesicles in the ME (12), suggesting that OPCs can regulate glutamatergic synapse numbers in response to dietary signals.

Furthermore, oligodendrocyte lineage cells in the ME have been shown to express an array of genes related to extracellular matrix (ECM) deposition and remodelling. In particular, pre-OLs are enriched for a glycoprotein called tenascin R, which can be visualised using *Wisteria floribunda* agglutinin (WFA), and which is upregulated following short-term fasting and refeeding (12). Genetic blockade of OPC differentiation results in loss of this WFA+ ECM network and, in response to fasting only, a protease called *Adamts4* is upregulated in the oligodendrocyte lineage. Overall, there is compelling evidence showing that OPCs are highly sensitive to dietary signals, to which they can respond by remodelling the ME ECM. However, it remains to be seen whether isolated modulation of the ME ECM network would result in changes in the function of LepR+ neurons and, in turn, alter feeding behaviour so as to respond to dietary signals (3).

## Microglia

Microglia can be considered to be the ‘sentinels’ of the CNS, capable of sensing the presence of invading pathogens and acting as the brain’s innate immune system. Microglia are typically grouped into two different states: resting or activated, which are morphologically and molecularly distinct (47). Unlike neurons and other glia (or macroglia) in the CNS, microglia are not derived from the neuroectoderm. Instead, microglia have been shown, in mice, to originate from the yolk sac and then to traverse the embryonic vasculature to colonize the neural tube by around E9.5 (48). In recent years, functions of microglia that are distinct from their role as the brain’s innate immune system have been described. These functions include supporting axon guidance, synaptic patterning, regulating myelination, and controlling the genesis and death of multiple other cell types through secreted factors, direct contact, and often, through phagocytosis (49).

With regards to the NES, evidence suggests that microglia could regulate aspects of the development and function of the hypothalamus (50). Microglia are present throughout the structure of the hypothalamus, including the ME (3, 51) and, during development, they regulate the size of populations of OPCs (43, 44) and other glia, including in the hypothalamus (52).

Furthermore, a growing body of work indicates that microglia directly respond to various hormones, including sex steroids (53) and neurohormones such as corticotropin-releasing factor (CRF or CRH) (54), which may regulate proliferation, apoptosis and expression of inflammatory markers in microglia (55, 56). While their sensitivity to such hormones does not demonstrate an involvement in the NES, it does raise the question of whether microglia could possibly form part of established feedback loops regulating the output of the gonadotropic and hypothalamic-pituitary-adrenal (HPA) axes. Furthermore, given the known role of microglia in surveillance of the contents of the extracellular milieu (57), microglia would seem to be a prime candidate cell type for dynamically regulating the function of the ME in response to changes in circulating substances, and could conceivably do so in the ME by phagocytosing other cells (44) or by pruning synapses (58). Interestingly, microglia have already been observed to interact with and phagocytose nerve terminals in the posterior pituitary (59), leading to the question of whether microglia could similarly perform synaptic pruning in the ME.

Finally, a high fat diet-induced mouse model of obesity has been shown to have increased microglia cell numbers in the hypothalamus (15, 60). Use of the chemotherapeutic cytosine arabinoside (Ara-C) resulted in reduced microglia and reduced expression of inflammatory markers, which were associated with reduced adiposity, feeding, body weight, and increased sensitivity to leptin (15). This finding has led to a postulated model of diet-induced hypothalamic inflammation that contributes to obesity (61). However, future work should aim to further understand causal relationships between OPCs, microglia and leptin-sensing in the ME, given that OPCs are also ablated by Ara-C treatment and may provide trophic support to LepR+ dendrites in the ME (3). Indeed, specific reduction of microglia in the brain *via* the CSF1R inhibitor PLX3397 was not sufficient to alter body weight in normal mice (3, 60). However, a role of increased microglia numbers and a pro-inflammatory environment in the ME in models of obesity is clear, given that PLX3397 reduces body weight and overeating in the context of high fat diet (60).

## Astrocytes

Astrocytes are a type of glia with a characteristic stellate shape that are found throughout the CNS. Astrocytes serve a wide variety of roles, including providing trophic support to neurons, regulating extracellular ion- and nutrient composition, glucose sensing, tissue repair and maintaining BBB function (62). As a population, astrocytes are morphologically, functionally, and molecularly heterogenous (13, 63). Astrocytes present in the hypothalamus differ from those found in other brain regions (64), for example, in that they lack glutamate uptake currents (65) and exhibit a differential response to ErbB receptor activation (66). A substantial body of work already exists demonstrating that astrocytes respond to many hormonal signals including sex steroids, glucocorticoids, and thyroid hormones, which would suggest they could potentially form part of NES feedback loops (13). Also, the well-established ability of astrocytes to sense and transport glucose would, in particular, implicate them in modulating the neuroendocrine response to nutritional signals. Indeed, in mice, following refeeding after a 24 hour fast, astrocytes in the ME were the most responsive cell types in terms of differential gene expression (12).

Within the hypothalamus, the functional interaction between glia including astrocytes, and neurons has been extensively investigated [see reviews (67, 68)]. However, relatively little is known about the role of astrocytes at the level of the ME. It is clear that the ME contains astrocytes and, as mentioned, astrocytes in the ME are highly responsive to dietary signals (12). Astrocytes may also have an immune-regulatory function in the ME. Astrocytes in the ME have been reported to become immunoreactive for IL-6 in response to lipopolysaccharide (LPS) injection in mice (69). However, whether astrocytes play a role in regulation of NES output at the level of the ME is poorly understood, and most hypotheses regarding functions of astrocytes in the ME are extrapolated from what is known about astrocyte-neuronal interactions in the wider hypothalamus (67, 68) (**Table 1**).

For example, it is known that astrocytes interact with the cell bodies of GnRH-expressing neurons (14) and evidence suggests that activation of astrocytic and/or tanycytic erbB1 receptors by TNFα leads to the secretion of prostaglandin E_2_ which in turn stimulates the release of GnRH from neurons into the bloodstream. Furthermore, oestradiol seems to increase the rate of astrocytic TNFalpha/erbB1 (TNFα/erbB1) signaling events (10, 70), suggesting that the oestrous cycle or other physiological causes of oestrogen fluctuations could feedback into GnRH neuronal function through astrocytes. In principle, a similar regulation could be exerted at the level of the ME, though this has not yet been tested.

Finally, it had been demonstrated that hypothalamic astrocytes sense blood glucose and express receptors for some neurohormones as well as for leptin and ghrelin. Fasting also triggers astrocytic plasticity around agouti-related peptide (AgRP)-expressing neurons, thereby removing inhibitory signals and increasing AgRP neuronal activation through release of prostaglandin E_2_ (71), in a way that is similar for GnRH+ neurons mentioned above. Therefore, there is a clear involvement of astrocytes in dynamically responding to dietary signals (13), which would align with the demonstratable sensitivity of ME astrocytes to fasting/refeeding in mice (12). However, a targeted approach for studying the role of astrocytes in the ME is required.

## Conclusions and Future Perspectives

There is little doubt that the ME represents a locus of the NES through which profound regulatory control is leveraged in response to dynamic signals, such as diet and hormones. The centrality of the NES to wide aspects of human physiology, health, but also ageing, makes investigations into the ME and its constituent cell types worthwhile, both in efforts to understand physiology and in treating disease. Increasing understanding of the cell and molecular biology of tanycytes, astrocytes, microglia and oligodendrocyte lineage cells (**Table 1**) therefore promises to be a highly fruitful avenue of research in identifying disease aetiology, new drug targets, and new therapies.

Therefore, the field is now faced with the problem of dissecting out the individual functions of the different glial cell types in the ME. Given the tight spatial constraints within the ME, complex interplay between the various glial elements resident in the ME seems highly likely (**Figure 1**), and it is difficult to categorically claim that a function, disease or phenotype is due to a particular cell type. For example, a role in the leptin system has been described for tanycytes (8, 11, 26), OPCs (3) and microglia (3, 15), and it seems likely that differentiating OPCs and oligodendrocytes have a role to play here as well, by maintaining the ECM composition of the ME (12). Future research must use a holistic approach that investigates not only glial-neuronal interactions but also glia-glia interactions, and a wide-range of genetic and experimental tools are available to study the biology of glia both *in vivo* and *in vitro* (72).

A further open question is to what extent regulation of NES function is mediated through self-contained systems within the ME. To date, most works investigating functional roles of glia in the NES have shown a requirement for communication between the ME and wider hypothalamus, such as in the tanycytic regulation of POMC neurons in the ArcN (27), or requirement of OPCs for maintaining sensitivity of neurons to leptin (3). Alternatively, perhaps glia at the level of the ME respond directly to peripheral and hormonal signals in such a way as to change the structure and function of the ME itself and, in turn, the function of the NES.

Finally, while in this review we have focussed on glia, the ability of neuronal elements to shape and regulate the function of the ME should not be overlooked. For example, it was recently shown that MCH-expressing neurons, like tanycytes, regulate permeability of the ME capillaries by secreting VEGF (73). We also have not discussed other non-neuronal elements, such as endothelial cells, pericytes, and immune cells other than microglia. For example, endothelial cells in the ME express Sema3A in an oestrogen-dependent manner that is associated with axonal outgrowth of GnRH+ neurons. Selective ablation of Sema3A from endothelial cells reduces ovulatory luteinizing hormone (LH) secretion, suggesting a functional deficit in the ME (74). Being outside of the blood brain barrier (BBB) it is possible for other circulating immune cells to be found in the ME, as well as microglia. Indeed, the perivascular space of ME capillaries is populated with macrophages, and infiltration of the ME with macrophages in high-fat diet and obesity in mouse models may contribute to diet-induced hypothalamic inflammation (75–77). Pericytes may also able to dynamically respond to peripheral signals and regulate ME function. In the medio-basal hypothalamus, leptin has been shown to be up-taken by pericytes, which exhibit calcium fluctuations in response to leptin increase. Loss of LepR in pericytes results in increased food intake and increased body weight and fat mass, which may result from altered paracellular permeability (78). Given the known presence of LepR+ dendrites in the ME, it may be reasonable to postulate that at least part of the observed changes in feeding behaviour and body weight seen due to loss of pericyte-expression of LepR (78) is due to a role of leptin regulating pericyte-mediated permeability of ME capillaries.

## Author Contributions

RC and CG wrote the initial review. RC, CG and RL-B contributed to the critical reading of the review. All authors contributed to the article and approved the submitted version.

## Funding

This work was supported by the Francis Crick Institute, which receives its core funding from Cancer Research UK (FC001107), the UK Medical Research Council (FC001107), and the Wellcome Trust (FC001107). For the purpose of Open Access, the author has applied a CC BY public copyright license to any Author Accepted Manuscript version arising from this submission. This work was also supported by an additional UK Medical Research Council grant (reference MR/T000759/1).

## Conflict of Interest

The authors declare that the research was conducted in the absence of any commercial or financial relationships that could be construed as a potential conflict of interest.

## Publisher’s Note

All claims expressed in this article are solely those of the authors and do not necessarily represent those of their affiliated organizations, or those of the publisher, the editors and the reviewers. Any product that may be evaluated in this article, or claim that may be made by its manufacturer, is not guaranteed or endorsed by the publisher.
